# Macaque antibodies targeting Marburg virus glycoprotein induced by multivalent immunization

**DOI:** 10.1128/jvi.00155-24

**Published:** 2024-06-04

**Authors:** Benjamin M. Janus, Ruixue Wang, Thomas E. Cleveland, Matthew C. Metcalf, Aaron C. Lemmer, Nydia van Dyk, Sarah Jeong, Anagh Astavans, Kenneth Class, Thomas R. Fuerst, Gilad Ofek

**Affiliations:** 1 Department of Cell Biology and Molecular Genetics, University of Maryland, College Park, Maryland, USA; 2 Institute for Bioscience and Biotechnology Research, University of Maryland, Rockville, Maryland, USA; 3 Biomolecular Measurement Division, National Institute of Standards and Technology, Gaithersburg, Maryland, USA

**Keywords:** Marburg virus, filovirus, glycoprotein, monoclonal antibodies, neutralizing antibodies, immunization, macaque, multivalent

## Abstract

Marburg virus infection in humans is associated with case fatality rates that can reach up to 90%, but to date, there are no approved vaccines or monoclonal antibody (mAb) countermeasures. Here, we immunized Rhesus macaques with multivalent combinations of filovirus glycoprotein (GP) antigens belonging to Marburg, Sudan, and Ebola viruses to generate monospecific and cross-reactive antibody responses against them. From the animal that developed the highest titers of Marburg virus GP-specific neutralizing antibodies, we sorted single memory B cells using a heterologous Ravn virus GP probe and cloned and characterized a panel of 34 mAbs belonging to 28 unique lineages. Antibody specificities were assessed by overlapping pepscan and binding competition analyses, revealing that roughly a third of the lineages mapped to the conserved receptor binding region, including potent neutralizing lineages that were confirmed by negative stain electron microscopy to target this region. Additional lineages targeted a protective region on GP2, while others were found to possess cross-filovirus reactivity. Our study advances the understanding of orthomarburgvirus glycoprotein antigenicity and furthers efforts to develop candidate antibody countermeasures against these lethal viruses.

**IMPORTANCE:**

Marburg viruses were the first filoviruses characterized to emerge in humans in 1967 and cause severe hemorrhagic fever with average case fatality rates of ~50%. Although mAb countermeasures have been approved for clinical use against the related Ebola viruses, there are currently no approved countermeasures against Marburg viruses. We successfully isolated a panel of orthomarburgvirus GP-specific mAbs from a macaque immunized with a multivalent combination of filovirus antigens. Our analyses revealed that roughly half of the antibodies in the panel mapped to regions on the glycoprotein shown to protect from infection, including the host cell receptor binding domain and a protective region on the membrane-anchoring subunit. Other antibodies in the panel exhibited broad filovirus GP recognition. Our study describes the discovery of a diverse panel of cross-reactive macaque antibodies targeting orthomarburgvirus and other filovirus GPs and provides candidate immunotherapeutics for further study and development.

## INTRODUCTION

Filoviruses are enveloped non-segmented negative-sense single-stranded RNA viruses that cause severe hemorrhagic fever disease in humans with case fatality rates reaching up to 90% (1–6). Viruses belonging to two genera of the *Filoviridae* family, *Orthoebolavirus* and *Orthomarburgvirus*, have caused outbreaks in humans. These include the orthoebolaviruses Ebola (EBOV), Sudan (SUDV), and Bundibugyo (BDBV), and the orthomarburgviruses Marburg (MARV) and Ravn (RAVV) (7–9). In the case of Marburg viruses, which were the first filoviruses documented to emerge in humans in 1967, re-emergence has occurred intermittently since then with notable outbreaks in the Democratic Republic of the Congo (DRC) between 1998 and 2000 that led to 154 cases and 128 deaths and in Angola between 2004 and 2005 that led to 252 cases and 227 deaths. More recently, Marburg virus re-emergence was reported in Guéckédou Guinea in 2021, in the Ashanti region of Ghana in 2022, and in Equatorial Guinea and Tanzania in 2023, regions that in some cases had not previously reported a single case of the virus (8). The sporadic geographical settings and timings of orthomarburgvirus outbreaks have highlighted the unpredictable nature of their emergence and the need for effective clinical countermeasures.

Attachment and entry of orthomarburgviruses to host cells is mediated by their surface glycoprotein, GP, which is made up of two disulfide-linked subunits, GP1 and GP2. While GP1 mediates interactions with host cell entry receptors, GP2 anchors the glycoprotein to viral membrane and mediates membrane fusion with host cell membranes. Both GP1 and GP2 are antigenic targets for neutralizing and protective antibodies (10–19). On GP1, the predicted receptor binding region (RBR) is the main target of neutralizing antibodies (nAbs), while the mucin-like domain is the target of non-neutralizing but protective antibodies that are hypothesized to inhibit viral infection by preventing viral release from infected cells (10, 11, 15). On GP2, a region at its N-terminus referred to as the “wing”, is targeted by protective but weakly or non-neutralizing antibodies that are thought to protect by recruitment of immune effector cells (17, 18). A region at the base of GP has also been recently reported as a target for neutralizing antibodies (19). Although sources for effective mAbs against orthomarburgviruses have included both human survivors of natural infection and immunized animals, to date, isolation of RBR-directed neutralizing antibodies has only been reported from human survivors, suggesting gaps may exist in current immunization or antibody isolation strategies (11, 16). While mAb protection studies in nonhuman primates (NHPs) have shown some degree of success, in particular using RBR-directed neutralizing antibodies, high antibody doses and administration by 5 days post-infection appear to be required for protection (20).

Immunization with multivalent combinations of antigens has been utilized in various contexts to expand immunological breadth against viral antigens, both in vaccine development and therapeutic mAb development (21–23). Multivalent immunizations not only offer the potential for induction of autologous immune responses against immunized species but also for induction of heterologous immune responses against conserved epitopes within a virus *family* or *genus*, including potentially against member species that have yet to emerge. In the case of filoviruses, multivalent immunization platforms based on a variety of GP immunogens have been reported, including recombinant GP protein subunits, replication-competent or deficient viral vectors expressing filovirus GPs, virus-like particles (VLPs), nucleic acids, or combinations thereof (21, 24–36). Administered immunization regimens have generally involved concomitant single-dose or multi-dose immunizations with cocktails of filovirus antigens, or alternatively, sequential heterologous prime-boost strategies. Both approaches have almost invariably resulted in induction of protective polyclonal responses against autologous species, and in some cases against heterologous filoviruses as well (21, 24–26, 29–31, 33, 37–41).

With the goal of inducing both autologous and heterologous antibodies against orthomarburgviruses, and against filoviruses more broadly, we immunized Rhesus macaques with heterologous prime-boost combinations of antigens belonging to species MARV, SUDV, and EBOV. Immunogens included VLPs composed of GP, VP40, and NP and recombinant GP ectodomains with or without intact mucin-like domains. Immunization regimens were weighted with MARV and SUDV antigens to augment immune responses against these species. Serological analyses revealed induction of both autologous and heterologous serum antibody binding and neutralization titers in all animals. Memory B cells from the animal that developed the highest titers of MARV neutralizing antibodies were subsequently sorted using a heterologous orthomarburgvirus GP probe to recover a panel of cross-reactive antibodies. We describe the functional characterization of this panel herein, including antibodies with pan-filovirus reactivity and potent neutralizing antibodies that target the RBR, representing the first reported isolation of orthomarburgvirus RBR-directed neutralizing antibodies from animal immunizations.

## MATERIALS AND METHODS

### Filovirus antigens

Virus-like particles composed of filovirus GP, NP, and VP40 of species MARV (Musoke), SUDV (Yambio), and EBOV (Mayinga) and recombinant GPΔTM and GPΔMuc antigens corresponding to species MARV (Angola), SUDV (Yambio), and EBOV (Mayinga) were purchased from an outside vendor (Integrated Biotherapeutics, Gaithersburg, MD) (30). The GPΔTM antigens included full GP ectodomains covering residues 1–627 of EBOV and SUDV GP, and residues 1–636 of MARV GP. GPΔMuc antigens were comprised of GP residues 1–311 fused to residues 464–637 for EBOV and GP residues 1–313 fused to residues 474–640 for SUDV. Recombinant BDBV and RAVV glycoproteins were expressed in HEK293 cells, as previously described (38).

### Sequence identity matrices

GP full-length sequence identity matrices were calculated in Bioedit using orthomarburgvirus strains MARV Musoke YP_001531156.1, MARV Angola Q1PD50.1, and RAVV Ravn YP_009055225.1, and orthoebolavirus strains EBOV Mayinga AAN37507, SUDV Yambio ABY75325, and BDBV AGL73460.1.

### Animal immunizations

All animal studies were undertaken at Advanced Bioscience Laboratories (ABL, Rockville, MD) through their subcontractor Bioqual (Rockville, MD). Bioqual’s facilities were fully accredited by the Association for Assessment and Accreditation for Laboratory Animal Care International (AAALAC #624). Veterinary care was administered in accordance with The Guide for the Care and Use of Laboratory Animals, the Animal Welfare Act as amended, the PHS Policy on Humane Care and Use of Laboratory Animals, and all applicable local, state, and federal laws. All studies were approved by the University of Maryland and the ABL/Bioqual Institutional Animal Care and Use Committees (project #918366).

Three Rhesus macaques (two females and one male) of the species *Macaca mulatta* of Chinese origin were immunized intramuscularly 4 times at 2-week intervals. Animals were ~5 years of age and weighed between 4.04 and 5.86 kg. MARV VLP prime immunizations were administered at 1 mg doses. MARV and SUDV VLP bivalent boosts were administered at 0.5 mg doses for each species. All recombinant GP ectodomain antigens were administered at 100 mcg for each species. All immunizations were formulated in 0.5 mL TiterMax Gold adjuvant (Sigma-Aldrich Inc., St. Louis, MO). Bleeds were conducted on day 7 after each inoculation to collect serum and peripheral blood mononuclear cells (PBMCs).

### ELISA assays

Nunc MaxiSorp 96-well ELISA plates (Thermo Fisher Scientific Inc., Waltham, MA) were coated with filovirus GPΔMuc proteins at 4°C overnight. Plates were washed in PBS pH 7.4 containing 0.05% Tween 20 and then blocked in PBS pH 7.4, 5% fetal bovine serum, and 2% non-fat dry milk powder for 1 hour at room temperature. The plates were washed and then incubated with fivefold serial dilutions of either monoclonal antibody or serum starting at 10 µg/mL or 1/10 dilution, respectively, for 1 hour. Plates were washed and a 1/2,500 dilution of horseradish peroxidase-conjugated goat anti-human secondary antibody (Jackson Immunoresearch, West Grove, PA) in blocking buffer was added for 1 hour. After washing, ELISAs were developed with TMB ELISA substrate solution (Bio-Rad Laboratories, Inc., Hercules, CA) and stopped using 1N sulfuric acid. Plates were read at an absorbance of 450 nm.

Competition ELISAs were undertaken by coating half-area ELISA plates (Greiner Bio-One, Monroe, NC) with 1 µg/mL of the benchmark antibodies at 4°C overnight. The next day, plates were washed in a wash buffer containing PBS pH 7.4 supplemented with 0.05% Tween 20 and then blocked in PBS pH 7.4, 5% fetal bovine serum, and 2% non-fat dry milk powder for 1 hour at room temperature. During this time in a separate non-binding U well shape plate (Greiner Bio-One, Monroe, NC) competing antibody was diluted to a final concentration of 5 µg/mL in blocking buffer and added to 2 µg/mL GP that was biotinylated through a fused Avi-tag (Avidity, Aurora, Colorado) and incubated for 1 hour at room temperature. The GP antibody mixture was then added to ELISA plates coated with capture antibody and incubated for 1 hour at room temperature. Plates were washed in wash buffer and then incubated with a 1:10,000 dilution of goat anti-biotin antibody (Thermo Fisher Scientific, Waltham, MA) in PBS pH 7.4 supplemented with 0.05% tween-20. The ELISA was developed as described above.

### Pseudovirus production

The generation of Murine Leukemia Virus (MLV)-based pseudoviruses with different filovirus GPs was carried out as previously described (42). Briefly, codon-optimized full-length genes of wild-type EBOV GP (GenBank: AAN37507.1), SUDV GP (GenBank: ALL26375.1), BDBV GP (GenBank: AGL73460.1), MARV GP (GenBank: YP_001531156.1), and RAVV GP (Genbank: Q1PDC7.1) were synthesized and constructed into a pCDNA3.1(-) expression vector using XbaI and HindIII (GenScript, Piscataway, NJ). The pseudoviruses were then produced by co-transfection of the human embryonic kidney 293T (HEK 293T) cells with the MLV Gag-Pol packaging vector (kindly provided by Dr. Jonathan K. Ball, the University of Nottingham), the Luciferase reporter plasmid (kindly provided by Dr. Jonathan K. Ball), and the GPs of five filoviruses constructs using Lipofectamine 3000 (Thermo Fisher Scientific, Waltham, MA) by following the manufacturer’s protocols. No-envelope control (empty plasmid) was used as a negative control in the experiments. After 6 hours, the medium was replaced by fresh DMEM with 10% FBS. At 48 hours and 72 hours after transfection, the culture supernatants containing filoviral pseudoviruses were harvested, passed through 0.45-μm pore-size filters, and used to infect target cells. Luciferase activity was detected using BrightGlo (Promega, Madison, WI) and expressed as relative light units (RLU) to determine the dilution used in neutralization assays.

Production of Vesicular Stomatitis Virus (VSV)-based filovirus GP pseudoviruses was performed according to the manufacturer’s instructions (Kerafast, Boston, MA). Briefly, HEK293T cells transfected with respective full-length filovirus GPs were transduced with VSV∆G-G for 2–4 hours (43). Cells were washed twice with PBS 7.4 and grown in DMEM containing 1.5% FBS and 1% Pen-Strep for 24 hours at 37°C and 5% CO_2_. VSV∆G-GP supernatants were collected and centrifuged for 10 minutes at 300 × *g* and passed through a 0.45-µm filter.

### Pseudovirus neutralization

To test animal sera for MLV or VSV-based pseudovirus neutralization, Vero E6 cells (ATCC, Manassas, VA) were pre-seeded into 96-well plates at minimum densities of 1 × 10^4^ cells per well and grown overnight at 37°C and 5% CO_2_ in DMEM containing 10% FBS and 1% Pen-Strep. The next day, pseudoviruses were incubated with defined concentrations of heat-inactivated serum at serial dilutions for 1 hour at 37°C and then added to each well. For MLV pseudovirus assays, the plates were incubated in a CO_2_ incubator at 37°C for 5 hours, followed by replacement of the mixtures with fresh medium and continued incubation for 72 hours at 37°C. For VSV pseudovirus assays, the plates were incubated for 1 hour at 37°C, 5% CO_2_ followed by the addition of an equal volume DMEM containing 5% FBS and 2% Pen-Strep, and continued incubation for 24 hours. To subsequently measure the degree of viral entry, luciferase activity in cell lysates was measured with the Bright-Glo Luciferase Assay System according to the manufacturer’s instructions (Promega, Madison, WI). Luciferase levels were measured using a FLUOstar Omega plate reader (BMG Labtech, Cary, NC) or a Tecan Spark 10M Plate reader (Tecan, Männedorf, CH). 50% and 80% inhibitory dilution (ID50 and ID80, respectively) titers were calculated as the serum dilution that led to a 50% or 80% reduction in relative light units (RLU) compared with pseudoviruses in control wells. ID50 and ID80 values were calculated through a dose-response curve fit with nonlinear regression plots using GraphPad Prism. All experiments involving the use of pseudoviruses were performed under biosafety level 2 conditions.

### Pseudovirus neutralization competition

The inhibition of the animal sera-mediated neutralization of MARV infection was tested using a neutralization inhibition assay (44) in which the macaque NHP1 sera (study day 49 and pre-immune) were preincubated for 30 minutes with purified RAVV GPΔMuc at concentrations of either 2.5 µg/mL or 25 µg/mL before the addition of the MARV pseudoviruses. After incubating for 1 hour at 37°C, the mixtures were then added to the 96-well plates of Vero E6 cells and further incubated for 5 hours before replacing them with fresh medium. With another 72-hour incubation, the luciferase activity was measured using the same method as described in the neutralization assays above. The inhibition effect of recombinant GP on MARV pseudovirus neutralization was reported as the change between the serum ID50 with or without the presence of the tested GP competitor. The neutralization inhibition efficiency was calculated based on the following calculation: [(percentage of neutralization w/o GPs - percentage of neutralization with GPs)/(percentage of neutralization w/o GPs)]x100. PBS was used as the negative control in the experiment.

### Antigen-specific memory B-cell sorting and mAb cloning

Macaque monoclonal antibodies were isolated by single B-cell cloning as previously described (45–47). In brief, macaque PBMCs were thawed and resuspended in staining media made up of RPMI 1640 supplemented with 10% fetal calf serum (FCS) at 37°. Cells were then washed in 10 mL staining media containing DNase I (Roche, Basel, Switzerland) and then resuspended in 100 µL of staining media containing 4 µg/mL of biotinylated RAVV GPΔMuc conjugated to streptavidin PE and 4 µg/mL of biotinylated RAVV GPΔMuc conjugated to streptavidin APC and incubated for 20 minutes. This was followed by the addition of a cocktail of CD3 APC-Cy7, CD8 APC-Cy7, Aqua Dead, CD14 Qdot 605 (BV605), IgM PE-Cy5, CD27 PE-Cy7, IgG FITC and CD20 PE-Alexa Fluor 700. Cells were gated for RAVV GPΔMuc double-positive B cells with a phenotype CD27+, IgG+, CD20+, Aqua Dead-, CD3-, CD8-, IgM- and sorted at single-cell precision on a BD FACSAria II (46). Individual cells were sorted directly into lysis buffer and then subjected to a reverse transcription-polymerase chain reaction (RT-PCR) using Superscript IV, as per the manufacturer’s guidelines (Thermo Fisher Scientific, Waltham, MA). Nested PCR using HotStarTaq (Qiagen, Hildent, Germany) was then used to amplify individual heavy and lambda/kappa light chains from the RT-PCR product. Heavy and light chain pairs were identified by agarose gel electrophoresis and sequenced by Sanger sequencing (Eurofins Genomics, Louisville, KY).

### Expression of antibodies and recombinant GP proteins

Antibody variable heavy and light chain regions were synthesized by gene synthesis with appended N-terminal signal sequences (Genscript Biotech, Piscataway, NJ) and subcloned into human IgG1 or lambda or kappa light-chain-based PCDNA3.1 mammalian expression plasmids. Plasmids were co-transfected into HEK-293F cells (ATCC, Manassas, VA) in FreeStyle Media using 293Fectin for transient protein expression (Thermo Fisher Scientific, Waltham, MA). Secreted IgGs were purified from cell supernatants with Protein A resin (Roche, Basel, Switzerland). IgGs were eluted at low pH using Protein A elution buffer (Thermo Fisher Scientific, Waltham, MA) and neutralized with Tris base pH 9.0. The IgGs were further purified by size exclusion chromatography (SEC) using an S200 column (Cytiva Lifesciences, Marlborough, MA) in PBS pH 7.4.

RAVV GPΔMuc fused with Hisx8, Strep II and Avi tags and a fibritin foldon trimerization domain was expressed in HEK293S GNTI^−/−^ cells (ATCC, Manassas, VA) using 293fectin transfection reagent and FreeStyle Media (Thermo Fisher Scientific, Waltham, MA). Supernatants were purified using Streptactin XT Resin purification (IBA Lifesciences, Göttingen, Germany). The GP protein was further purified by SEC Superdex 200 HiLoad 16/600 column in 150  mM NaCl, 2.5  mM Tris-Cl pH 7.5, and 0.02% NaN3. GP was biotinylated by the Avi tag (Avidity, Aurora, Colorado) and exchanged into PBS 7.4.

### Pepscan analysis

Peptide competitions were undertaken by incubating RAVV GPΔMuc at 200 ng per well in Nunc MaxiSorp 96-well ELISA plates (Thermo Fisher Scientific, Waltham, MA) overnight at 4°C. The plates were blocked as described above. In separate non-binding U-well-shaped plates (Greiner Bio-One, Monroe, NC), 200 ng of each of the overlapping 15-mer peptides across either GP1 or GP2 was incubated individually with 0.4 µg/mL of IgG for 1 hour at room temperature. 100 µL of each IgG peptide mixture was added to the plate with GP and incubated for 1 hour. Plates were washed and a horseradish peroxidase-conjugated goat anti-human secondary antibody (Jackson ImmunoResearch, PA) was added at a 1:2,500 dilution. Plates were washed and developed as above. Direct pepscan analysis was undertaken by binding 200 ng of each overlapping peptide to Nunc MaxiSorp 96-well ELISA plates (Thermo Fisher Scientific, Waltham, MA) overnight at 4°C. Plates were washed and blocked as above, and 0.4 µg/mL of IgG was then added to each well for 1 hour at room temperature. Plates were washed, and secondary antibodies were added as described above. Finally, plates were washed and developed as described above.

### Western blots

Mini-PROTEAN TGX gels were run and transferred to a PVDF or nitrocellulose membrane using the Turbo Blot protocol for mini-PROTEAN TGX gels (Bio-Rad Laboratories, Hercules, CA). The membranes were blocked for 5 minutes with 5% skim milk powder. The PVDF/nitrocellulose membranes were then divided into strips of one lane, each containing GP1 and GP2. The strips were placed in separate primary stain solutions with each antibody at 1 µg/mL. After staining on a platform rocker for 1 hour, the strips were washed three times for 5 minutes with TBST. All strips were then stained separately with a 1:5,000 dilution of goat anti-human IgG for 1 hour. After the secondary staining, the strips were again washed three times with TBST for 5 min. The strips were then placed together and developed by enhanced chemiluminescence (ECL) (Thermo Fisher Scientific, Waltham, MA) and imaged on a ChemiDoc imager (Bio-Rad Laboratories, Hercules, CA).

### Negative stain electron microscopy

Negative staining was performed following the optimized negative staining (OpNS) protocol as described (48). Optimized negative staining: a high-throughput protocol for examining small and asymmetric protein structure by electron microscopy (48). Briefly, complexes were diluted to 0.01 mg/mL and immediately applied to EM grids (Electron Microscopy Sciences #CF200-Cu). Grids were then incubated for 1 minute, blotted with filter paper, washed three times with water as described, and stained with fresh 1% uranyl formate solution for 30 s as described. The staining solution was then blotted with filter paper and grids were dried in a desiccator overnight prior to imaging.

Imaging was performed using a Talos Arctica (200 kV) system (Thermo Fisher Scientific) equipped with a Falcon 3EC detector. A nominal magnification of 73,000× was used, corresponding to a pixel size of 1.38 Å. Dose-fractionated movies were collected with a total dose of about 120 e/Å^2^, and motion correction was performed using RELION (49). Particle picking was done using crYOLO followed by 2D classification in RELION (49, 50).

### MARV-VSV pseudovirus release assay

HEK293T cells were seeded at approximately 70% confluence in 96-well plates and transfected with MARV Musoke GP using Lipofectamine 3000 (Thermo Fisher Scientific, Waltham, MA). The following day VSVΔG-G virus was added at 40 µL per well and incubated for 2 hours at 37°C and 5% CO_2_. Cells were then washed with DMEM containing 10% FBS, and antibodies pre-diluted at 50 µg/mL in 100 µL DMEM with 10% FBS added and incubated for 6 hours at 37°C and 5% CO_2_. The sample was harvested by diluting 10 µL of supernatant into 90 µL DMEM supplemented with 10% FBS followed by centrifugation at 1,000 × *g* for 5 minutes to remove cells. 90 µL of each sample was then added to VeroE6 cells seeded the prior day at 20,000 cells/well in 96-well plates. Plates were incubated overnight at 37°C and 5% CO_2_ and the following day were developed using BrightGlo (Promega, Madison, WI) following the manufacturer’s protocols. Virus titers were measured in relative luminescence units (RLU). Relative titers in the presence versus absence of antibodies were calculated using the formula: (RLU [with antibody]/RLU [without antibody])*100%. A two-tailed unpaired *t*-test was used in Graphpad Prism to assess statistical significance relative to the no-antibody controls.

## RESULTS

### Filovirus multivalent prime-boost immunization

**Fig 1:**
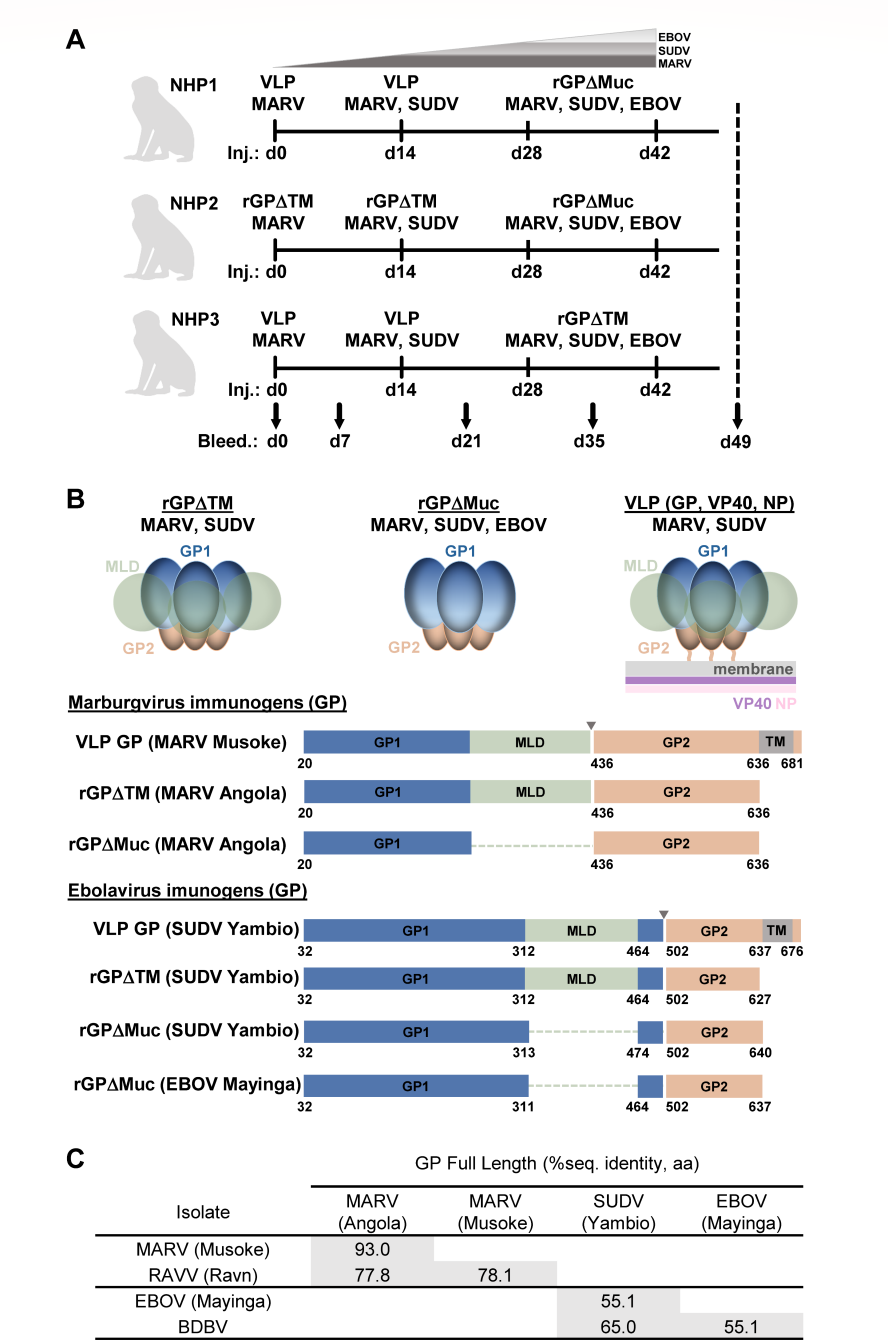
Multivalent prime-boost immunization of Rhesus macaques. (**A**) Immunization regimens for three Rhesus macaques, each receiving four immunizations at 2-week intervals. Serum bleeds were taken at day 0 (preimmune) and 7 days after each immunization. (**B**) Schematics of immunogens used in the study, including virus-like particles (VLPs) composed of GP, VP40, and NP, recombinant full-length GP ectodomains (GPΔTM), and recombinant GP ectodomains lacking mucin-like domains (GPΔMuc). (**C**) Intra-genus sequence identity matrix for full-length GPs corresponding to study immunogens.

Three Rhesus macaques of Chinese origin (two females and one male) were immunized with multivalent regimens made up of MARV, SUDV, and EBOV GP-based immunogens (Fig. 1A). To restrain possible immunodominance of EBOV GP (30) and enhance immune responses primarily against MARV GPs but also against SUDV GPs, a multivalent prime-boost approach weighted with MARV and SUDV immunogens was employed. All animals were primed exclusively with MARV-based immunogens, followed by bivalent boosts with MARV + SUDV immunogens, followed by two trivalent boosts with MARV + SUDV + EBOV immunogens (Fig. 1A). Animals were immunized a total of four times at 2-week intervals (study days 0, 14, 28, and 42) and bleeds were taken at day 7 after each vaccination, a timepoint shown to have a high frequency of antigen-specific plasmablasts (51, 52) (Fig. 1A). Immunogens included VLPs made up of GP, VP40, and NP, and recombinant GP ectodomains either with or without intact mucin-like domains (GPΔTM or GPΔMuc, respectively) (Fig. 1B). Recombinant glycoprotein immunizations were formulated in Titermax Gold adjuvant, a water-in-oil emulsion. While all SUDV and EBOV immunogens were based on the Yambio and Mayinga isolates, respectively, MARV immunogens were based on Musoke for the VLPs and Angola for the recombinant GPs, which further increased the antigenic breadth of immunized MARV variants. Intra-genus full-length GP sequence diversity for the corresponding immunogens was ~7% for the orthomarburgvirus antigens and ~45% for the orthoebolavirus antigens (Fig. 1C).

### Serum antibody binding to autologous and heterologous GPs

**Fig 2:**
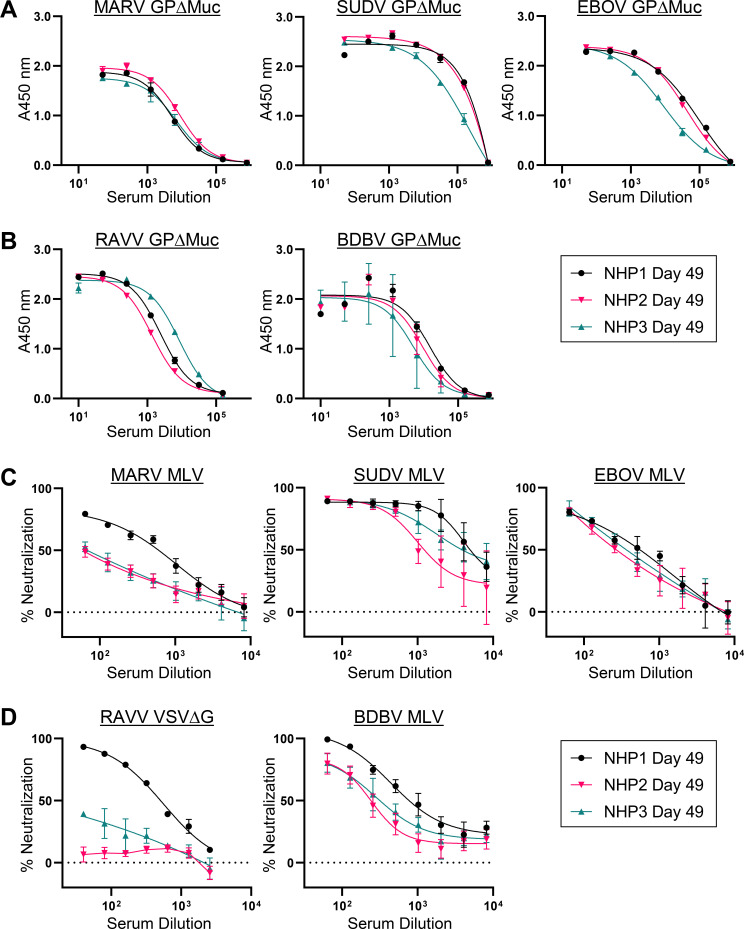
Autologous and heterologous serum antibody binding and neutralization titers. Study day 49 serum ELISA binding profiles for each animal to autologous (**A**) or heterologous (**B**) recombinant GPΔMuc proteins. Shown are means of technical duplicates with error bars indicating standard deviation. Study day 49 serum neutralization profiles for each animal against autologous GP pseudotyped MLV viruses (**C**) or heterologous GP pseudotyped MLV or VSVΔG viruses (**D**). Shown are means of technical duplicates with error bars indicating standard deviation. Results are of representative experiments repeated two or more times for orthomarburgvirus targets and 1–2 times for orthoebolavirus targets.

To assess serum IgG antibody binding titers elicited over the course of the study against autologous filovirus GP species, we tested serum bleeds taken 7 days after the first, second, and final boosts (study days 21, 35, and 49) for binding to recombinant MARV, SUDV, and EBOV GP∆Muc by ELISA. Serum IgG binding titers against all three autologous species were detected in all animals (Fig. 2; Fig. S1). Serum from day 49 terminal bleeds yielded 50% effective dilutions (ED50s) of binding to MARV, EBOV, and SUDV GP∆Muc that ranged from ~5.8 to 8.9 × 10^3^, ~9.0 to 98 × 10^3^, and 2.1 to 60 × 10^5^, respectively, with binding responses in animal NHP3 lagging behind those in NHP1 and NHP2 (Fig. 2A). Over the course of the study, responses against SUDV and EBOV GP∆Muc increased after the second and third boosts while those against MARV GP∆Muc were generally less responsive to these boosts (Fig. S1 and S2).

Since one of the objectives of the multivalent immunization approach was to induce immunological breadth against conserved regions within filovirus GPs, we also assessed serum antibody recognition of heterologous filovirus GPs. Terminal bleed day 49 serum from each of the three immunized macaques was tested by ELISA for recognition of heterologous RAVV and BDBV GPΔMuc proteins, which differ in sequence from their autologous full-length counterparts by up to 22.2% and 44.9%, respectively (Fig. 1C). The presence of IgG binding titers against RAVV and BDBV GPΔMuc was detected in sera from all three animals, with serum dilution ED50s ranging from 1.4 to 8.5 × 10^3^ and 5.0 to 14 × 10^3^, respectively (Fig. 2B). Serum reactivity against EBOV GPΔMuc was detected in day 21 serum from all three animals, a time point in the study preceding any EBOV GP immunizations, indicating the presence of heterologous binding titers against this species as well (Fig. S1 and S2). Taken together, our data confirmed that the heterologous prime-boost immunization approach employed in the study led to the successful elicitation of antibodies with both autologous and heterologous binding breadth.

### Serum antibody neutralization of autologous and heterologous pseudoviruses

We next tested terminal bleed serum (day 49) from all three animals for neutralization of Murine Leukemia Virus (MLV)-based viruses pseudotyped with autologous MARV Musoke, SUDV Boniface, or EBOV Mayinga GP. Animal NHP1, which received a MARV VLP prime, a MARV + SUDV VLP-based boost, followed by two trivalent boosts with MARV, SUDV, and EBOV GPΔMuc proteins, exhibited the highest overall IgG neutralization titers observed in the study against all three autologous viral species (Fig. 2C). Neutralization ID50s in this animal were observed at ~7.1 × 10^2^, ~5.6 × 10^3^, and ~2.0 × 10^3^, against MARV, SUDV, and EBOV pseudoviruses, respectively (Fig. 2C). Although neutralization titers against SUDV and EBOV were also observed in serum from animals NHP2 and NHP3, neutralizing titers against MARV in these animals were lower than those observed in NHP1 despite the presence of nearly equivalent levels of MARV GPΔMuc binding titers (Fig. 2A and C).

To determine whether heterologous neutralization breadth was induced in the animals, we next tested terminal bleed day 49 serum from each animal for neutralization of heterologous BDBV and RAVV pseudoviruses. As shown in Fig. 2D, neutralizing antibody titers against heterologous BDBV were observed in all three animals, with animal NHP1 serum yielding the highest heterologous neutralization potency of the three animals. Heterologous neutralizing titers against RAVV were tested using both VSV- and MLV-based pseudoviruses, revealing neutralizing titers mainly in NHP1 serum, with reduced or absent neutralizing responses in NHP3 and NHP2 sera, respectively (Fig. 2D; Fig. S3).

Taken together, these results indicated that the most pronounced neutralizing responses, against both autologous and heterologous viruses, were induced in NHP1, prompting us to further investigate the induced mAbs in this animal.

### Heterologous probe for isolation of cross-reactive B cells

**Fig 3:**
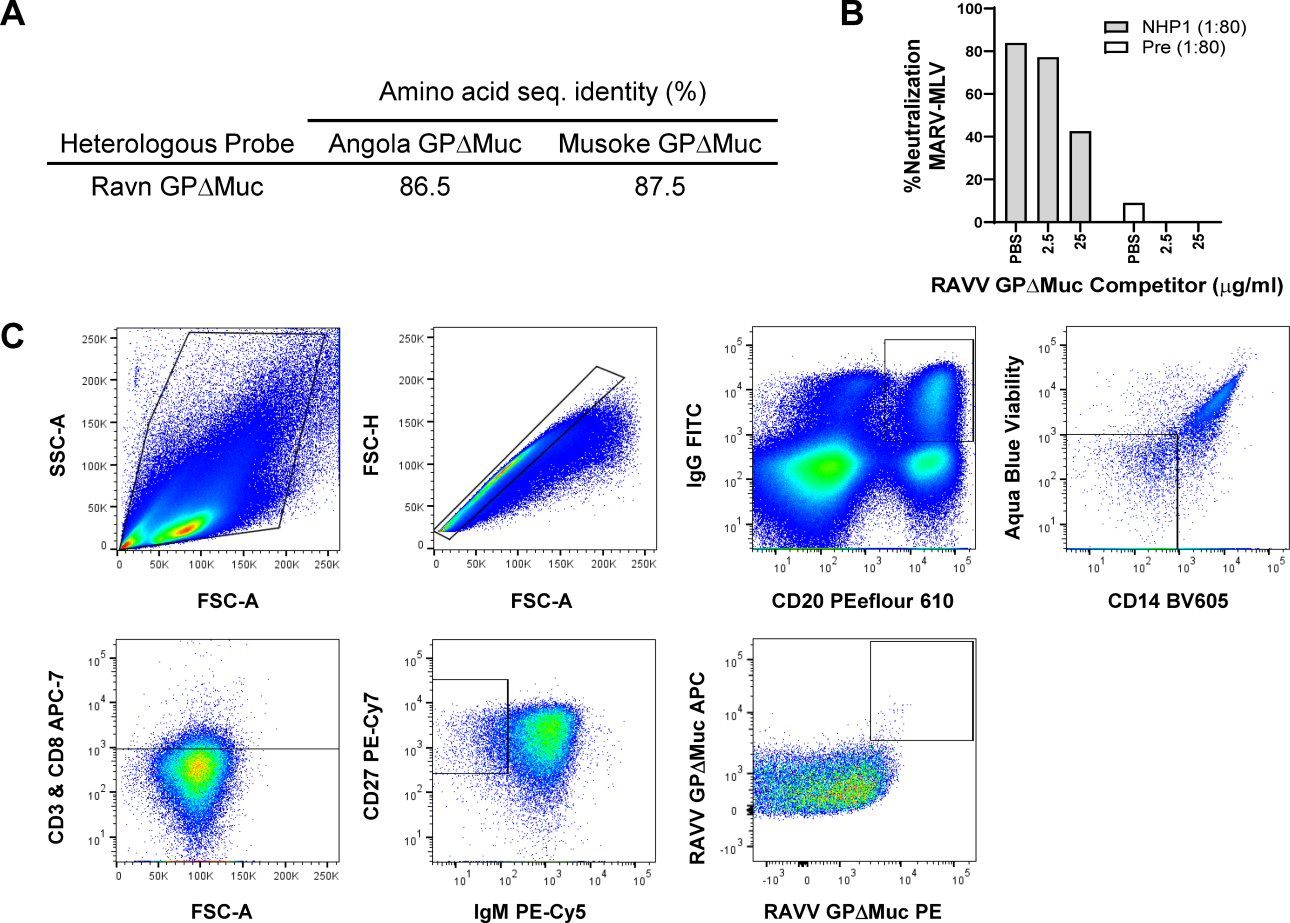
Antigen-specific memory B-cell sorting with a heterologous orthomarburgvirus GP probe. (**A**) Sequence divergence of the heterologous RAVV GPΔMuc B-cell sorting probe from autologous MARV Musoke and Angola immunogens. (**B**) Heterologous RAVV GPΔMuc probe competition for NHP1 day 49 serum neutralizing antibodies, against MARV-MLV pseudoviruses. Pre, preimmune. Shown are single replicates of a representative experiment repeated two times. (**C**) Memory B-cell sorting pipeline for RAVV GPΔMuc double-positive B cells with the phenotype CD27+, IgG+, CD20+, Aqua Dead-, CD3-, CD8-, CD14-, and IgM-.

To isolate cross-reactive antibodies, we developed a recombinant heterologous GP probe to select for cross-orthomarburgvirus reactive memory B cells from peripheral blood mononuclear cells (PBMCs) of animal NHP1. Toward this end, we utilized a fibritin foldon-trimerized heterologous RAVV GPΔMuc protein shown above to be recognized by NHP1 serum (Fig. 2B), which diverged in amino acid sequence from autologous Musoke and Angola MARV GPΔMuc by ~13% (Fig. 3A). To validate its use as a probe, we assessed its efficacy as a competitor in MARV-MLV pseudovirus neutralization assays, to gauge if it could be bound effectively by NHP1 serum heterologous neutralizing antibodies. Although the addition of the RAVV GPΔMuc probe at 2.5 µg/mL concentration to NHP1 serum prior addition to pseudoviruses and target cells was not sufficient to compete away serum neutralization, when added at 25 µg/mL it successfully reduced serum neutralization by ~60% (Fig. 3B). These results confirmed the presence of heterologous cross-orthomarburgvirus reactive neutralizing antibodies in NHP1 serum and validated the use of RAVV GPΔMuc as a heterologous probe for B-cell sorting.

### Antigen-specific memory B-cell sorting and monoclonal antibody cloning

**Fig 4:**
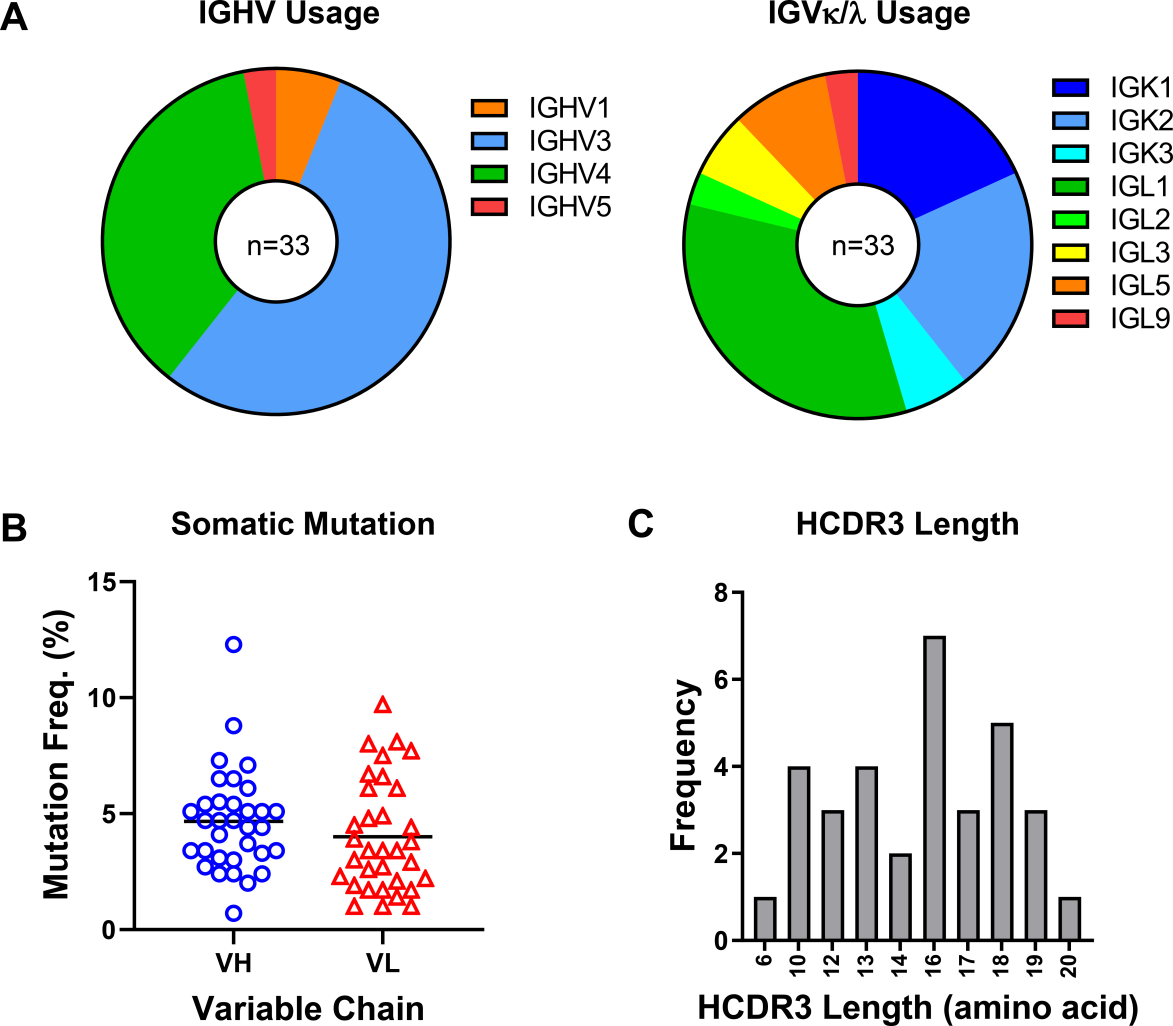
Antibody heavy and light chain sequence features. (**A**) Heavy and light chain gene usage of the 33 expressed monoclonal antibodies. (**B**) Heavy chain and light chain somatic mutation frequencies are shown as percentages of total amino acids in variable regions. (C) Heavy chain CDR3 length distribution across the antibody panel.

**Fig 5:**
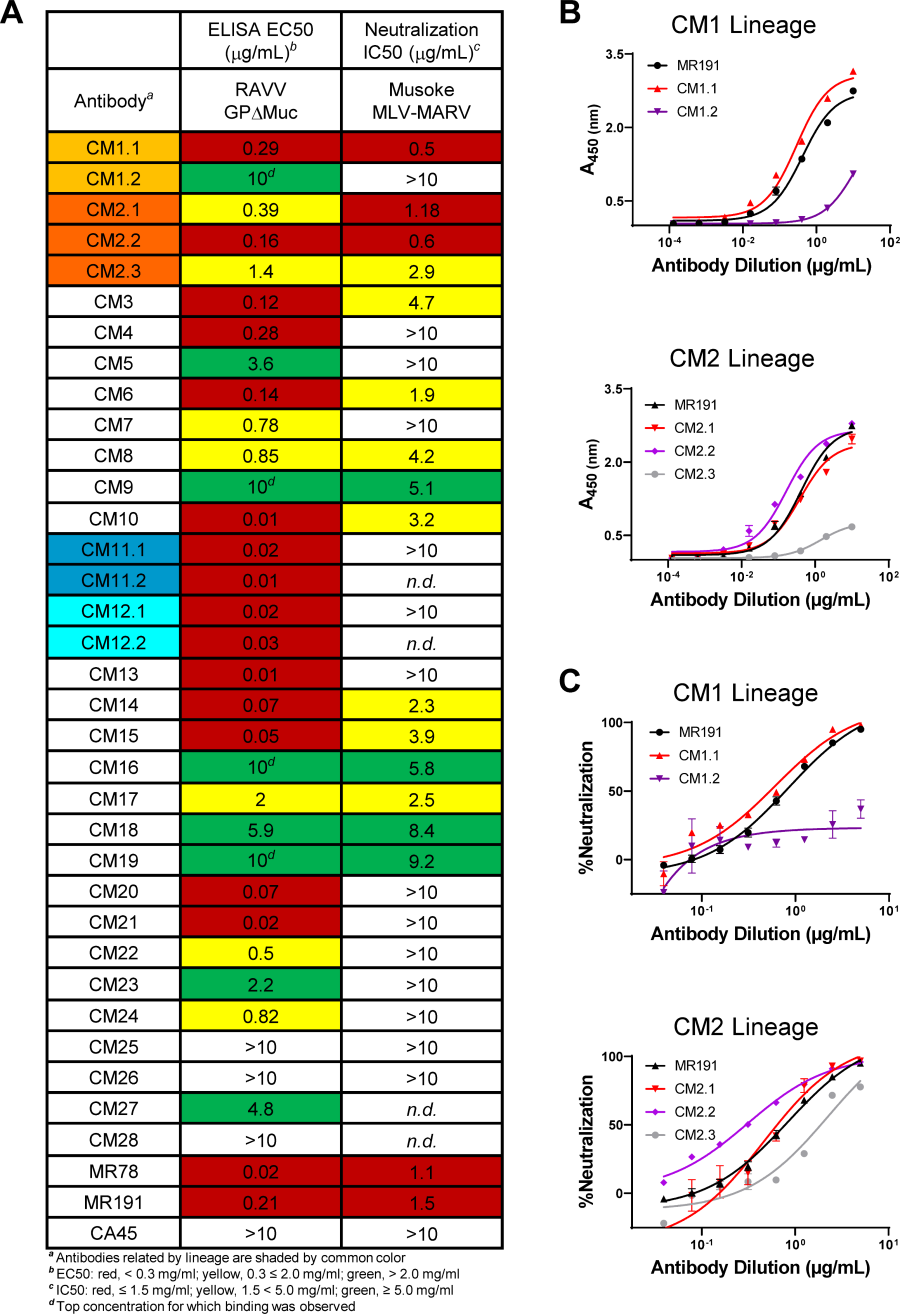
Antibody binding and pseudovirus neutralization. (**A**) Antibody ELISA binding EC50s to RAVV GPΔMuc and neutralization IC50s against MLV-MARV Musoke pseudoviruses calculated from individual plots shown in Fig. S4 and S5. Antibody CM1, CM2, CM11, and CM12 lineage variants are shaded light orange, orange, teal, and cyan, respectively. (**B**) ELISA binding profiles of antibody CM1 and CM2 lineage variants to recombinant RAVV GPΔMuc. Shown are means of technical duplicates with error bars indicating standard deviation. Results are of representative experiments repeated at least three times. (**C**) Neutralization of Musoke MLV-MARV pseudoviruses by antibody CM1 and CM2 lineage variants. Shown are means of technical duplicates with error bars indicating standard deviation. Results are of representative experiments repeated at least three times.

To isolate double-positive RAVV GPΔMuc reactive B cells, we stained NHP1 terminal bleed (day 49) peripheral blood mononuclear cells (PBMCs) with avi-tag biotinylated trimerized RAVV GPΔMuc protein conjugated with two types of fluorescently labeled streptavidin, APC and PE, along with a cocktail of reagents targeting memory B-cell surface markers. Our multi-color staining approach ensured the selection of B cells that were of the phenotype IgG^+^IgM^-^CD20^+^CD14^-^CD3^-^CD8^-^CD27^+^, RAVV GP^++^ (46, 53) (Fig. 3C). Of the ~480 B cells that were sorted into 96-well plates, we utilized the first 96-well plate to recover an initial panel of monoclonal antibodies through nested PCR amplification of heavy and light chain antibody variable regions, as previously described (46, 53). 58 out of 96 wells yielded successful amplification of both heavy and light chain antibody products that were subsequently sequenced. Based on a variety of sequence features, including sequence fidelity and completeness, immunogenetic diversity, the presence of lineage mates, HCDR3 loop length, and degree of somatic hypermutation, 34 mAb heavy and light chain pairs were selected for experimental characterization. The selected mAb sequences represented diverse immunogenetic backgrounds, corresponding to roughly 10 IGHV and 21 IGLV genes (Fig. 4A). A majority of the heavy chains were of VH3-background (Fig. 4A). Rates of somatic hypermutation ranged from 0.7% to 12.3% and 1.0% to 9.7% for heavy and light chains, respectively, while heavy chain CDR3 loop lengths ranged from 6 to 20 amino acids (Fig. 4B and C). A majority of the antibodies in the panel represented independent clonotypes, although nine variants belonged to one of four shared lineages (Fig. 5A). For experimental characterization, the heavy and light chain variable regions of the selected 34 mAbs were synthesized and subcloned into human IgG1 expression vectors for transient expression in HEK293 cells. Out of the 34 mAbs, 33 expressed to sufficient levels to permit further study.

### MAb binding to GP and pseudovirus neutralization

We assessed the binding of the 33 expressed mAbs to RAVV GPΔMuc by ELISA, alongside orthomarburgvirus GP-specific antibodies MR78 and MR191 and orthoebolavirus GP-specific antibody CA45 as controls (11, 31). 28 of the mAbs (representing 23 lineages) bound RAVV GPΔMuc with EC50 values that ranged from 0.01 to 10 μg/mL, confirming that the B-cell sorts led to successful isolation of orthomarburgvirus GP-specific mAbs (Fig. 5A; Fig. S4). Indeed, some of the antibodies bound with EC50 values that were commensurate or better than those observed for control antibodies MR78 and MR191 (Fig. 5A; Fig. S4).

Using a maximum antibody concentration of 10 µg/mL, we next tested the 28 GP-reactive mAbs for the capacity to neutralize MLV-MARV Musoke pseudoviruses (54). 16 of the 28 antibodies tested (~57%) exhibited neutralization of MLV-MARV to different degrees, with neutralization IC50 values ranging from 0.5 to 9.2 μg/mL (Fig. 5A; Fig. S5). Two of the antibody lineages, CM1 and CM2, exhibited the most potent neutralization observed in the panel with IC50s that ranged from 0.5 to 1.18 μg/mL, on par with IC50s obtained for the MR191 and MR78 controls (Fig. 5A and C; Fig. S5) (11). While some of the variants that belonged to the CM1 and CM2 lineages exhibited weak or undetectable neutralization, namely mAbs CM1.2 and CM2.3, such differences correlated with differences in binding capacity to recombinant GP by ELISA (Fig. 5A through C; Fig. S4 and S5). In contrast to their lineage mates, mAbs CM1.2 and CM2.3 also expressed at lower levels and were prone to proteolytic cleavage, consistent with potential biochemical instability. Nonetheless, our results confirmed that a majority of the mAbs in the panel effectively recognized heterologous RAVV GPΔMuc, with two of the lineages exhibiting highly potent MARV pseudovirus neutralization.

### Epitope mapping by overlapping pepscan analysis

**Fig 6:**
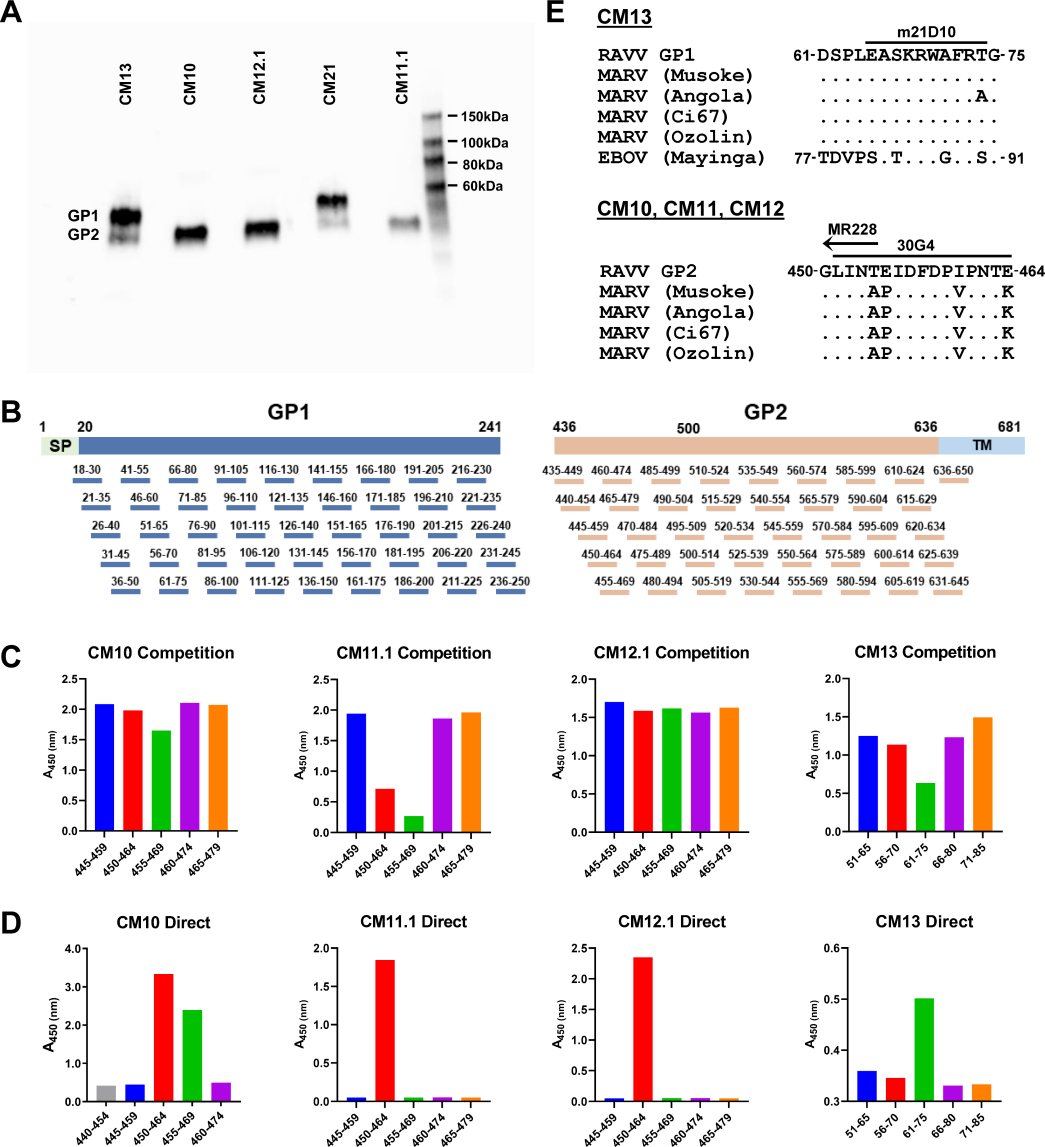
Epitope mapping by overlapping pepscan analysis. (**A**) SDS-PAGE Western blots of RAVV GPΔMuc probed with five mAbs observed to give detectable recognition of denatured GP. (**B**) Schematic of overlapping 15-mer peptides across the GP1 and GP2 subunits of RAVV GPΔMuc that were used for pepscan analyses. (**C**) Overlapping peptide ELISA binding competition for RAVV GPΔMuc recognition by mAbs CM10, CM11.1, CM12.1, and CM13, focused on regions of GP1 and GP2 that exhibited competition. Shown are single replicates of representative experiments performed 1–2 times. (**D**) Direct ELISA binding of mAbs CM10, CM11.1, CM12.1, and CM13 to overlapping peptides, focused on regions defined in *C*. Shown are single replicates of representative experiments performed two or more times. (**E**) Sequence alignments of the GP1 epitope of mAb CM13 across filoviruses, top, and of the GP2 epitope of mAbs CM10, CM11, and CM12 across orthomarburgviruses, bottom. Overlapping epitopes of previously characterized mAbs m21D10, 30G4, and MR228 are shown as bars above.

Prior to undertaking overlapping pepscan analysis for epitope mapping, we assessed whether any of the GP-reactive antibodies in the panel could recognize contiguous, non-conformational epitopes on RAVV GPΔMuc. Toward this end, RAVV GPΔMuc protein was applied to a denaturing SDS-PAGE gel and subjected to standard Western blotting procedures, using each individual GP-reactive antibody as a probe. Five of the tested mAbs gave detectable signals by Western blot analysis (Fig. 6A). Two mAbs, CM13 and CM21, reacted with a band corresponding to the size of GP1, while the remaining three mAbs, CM10, CM11.1, and CM12.1, targeted a band corresponding to the size of GP2 (Fig. 6A).

To further map the epitopes of these five Western-blot reactive mAbs, we generated a panel of overlapping 15-mer peptides covering the sequences of GP1 and GP2 of RAVV GPΔMuc, and undertook both competition and direct ELISA binding analyses (Fig. 6B through D). For mAbs CM10, CM11.1, and CM12.1, which were predicted to target the GP2 subunit, our analysis focused on binding to 46 overlapping peptides covering the GP2 ectodomain, spanning residues 435–650. To assess whether any of the 46 overlapping GP2 peptides could successfully compete for mAb recognition of RAVV GPΔMuc, we individually incubated each mAb with each peptide and then added the mixture to ELISA wells coated with RAVV GPΔMuc. These assays revealed that peptides 450–464 and 455–469 within the GP2 N-terminus (or “wing”), a region previously shown to be targeted by protective antibodies, successfully competed for CM10 and CM11.1 recognition of RAVV GPΔMuc (Fig. 6C and E) (17, 18). None of the peptides effectively competed for CM12.1 mAb recognition of RAVV GPΔMuc (Fig. 6C). To further verify CM10 and CM11.1 recognition of the GP2 N-terminus, and to also map the epitope of mAb CM12.1, we undertook direct ELISA binding analyses using the same set of overlapping peptides spanning the GP2 N-terminus (residues 440–479). mAbs CM10 and CM11.1 both bound peptide 450–464 directly, while mAb CM10 bound peptide 455–469 as well. Despite the inability of peptide 450–464 to effectively compete with RAVV GPΔMuc for CM12.1 recognition, direct binding of CM12.1 to peptide 450–464 was detected (Fig. 6C and D). Our results thus indicate that mAbs CM10, CM11.1, and CM12.1 all target an epitope within the GP2 N terminus, one that overlaps with epitopes of previously reported protective mAbs isolated from natural infection and animal immunizations (Fig. 6E) (11, 17, 18, 55).

To map the epitopes of GP1 Western-blot reactive mAbs CM13 and CM21, we employed a similar strategy but utilized a set of overlapping GP1 peptides instead (Fig. 6B). 45 overlapping 15-mer peptides spanning GP1 ectodomain residues 18–250 were used as competitors for CM13 and CM21 binding to RAVV GPΔMuc (Fig. 6B and C). Binding of CM13 to RAVV GPΔMuc was competed ~50% by a peptide spanning GP1 residues 61–75, although direct recognition of this peptide was weak (Fig. 6C and D). We note that peptide 61–75 lies in the vicinity of the predicted RBR on GP1, and partially overlaps with the epitope of a previously reported pan-filovirus reactive murine antibody, m21D10, one that was also isolated from multivalent immunization (Fig. 6E) (29). In contrast to CM13, none of the 45 overlapping 15-mer GP1 peptides competed with mAb CM21 for binding to RAVV GPΔMuc, nor were any recognized by direct ELISA (not shown), indicating other means will be necessary to map its epitope.

### Determination of antigenic binding competition groups

**Fig 7:**
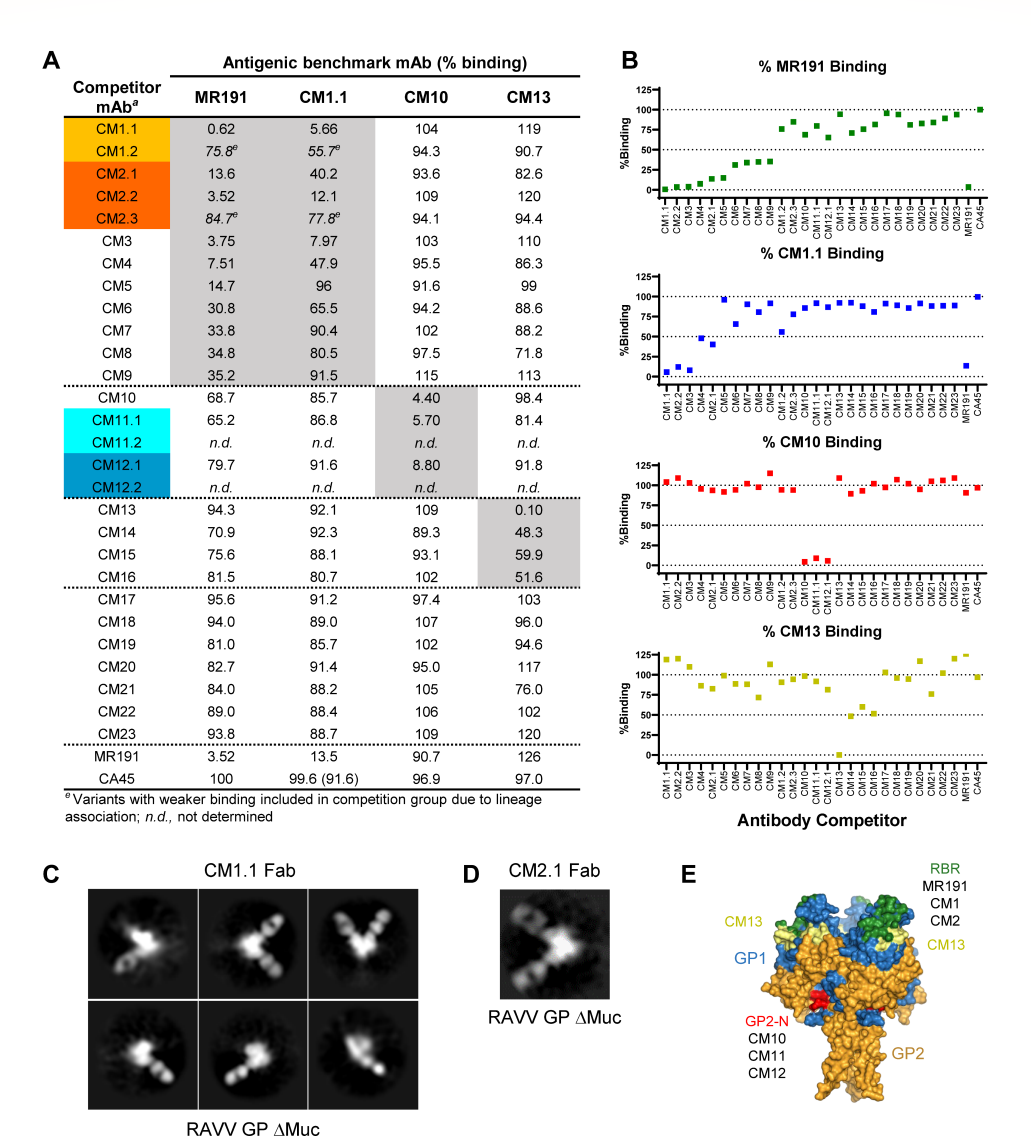
Antibody binding competition groups. (**A**) Shown are %-binding values of each antigenic benchmark mAb to biotinylated RAVV GPΔMuc in the presence of each competitor mAb. Percentages are calculated relative to the capture of biotinylated RAVV GPΔMuc in the absence of a competing mAb. Orthoebolavirus-specific mAb CA45 was used as a negative control. Results are of representative experiments performed 1–2 times with 1–2 replicates. (**B**) Plots of the %-binding competition against each benchmark mAb. (**C**) NSEM 2D class averages of CM1.1 Fab in complex with recombinant RAVV GPΔMuc protein. (**D**) NSEM 2D class average of CM2.1 Fab in complex with recombinant RAVV GPΔMuc protein. (**E**) Antigenic footprints of benchmark mAbs MR191, CM1.1, CM10, and CM13, mapped onto the surface of RAVV GPΔMuc (PDB ID 6BP2). GP1 and GP2 are colored blue and orange, respectively. The predicted RBR is colored green, based on the epitope of MR191. The first ordered residues within the N terminus of GP2 (GP2-N) are colored red and the CM13 epitope is colored yellow.

To further classify the antigenic targets of the antibodies in the panel, we undertook GP binding competition analyses to define antigenic competition groups. Four antibodies were selected as antigenic benchmarks for recognition of RAVV GPΔMuc against which all antibodies in the panel were tested as competitors. Benchmark mAbs included CM10 and CM13 that were mapped above to continuous epitopes on GP2 and GP1, respectively, along with two potent MARV neutralizing antibodies, CM1.1 from the present study and antibody MR191, a previously reported RBR-directed nAb (Fig. 7A and B) (11). Our binding competition assay entailed pre-incubation of each GP-reactive antibody in the panel with biotinylated RAVV GPΔMuc for 1 hour followed by the addition of the complex to ELISA plates pre-coated with each of the four antigenic benchmark antibodies. The degree to which the benchmark antibodies could capture biotinylated RAVV GPΔMuc alone or in the presence of competitor antibodies was assessed by detection with HRP-conjugated anti-biotin antibody.

As shown in Fig. 7A and B, roughly a third of the antibodies in the panel (nine lineages) fell within the RBR antigenic competition group in that they blocked between 64% to greater than 99% of MR191 binding to RAVV GPΔMuc. Two potent neutralizing antibodies, CM1.1 and CM2.1, and a subset of their lineage mates, also fell within this MR191 competition group (Fig. 7A and B). Indeed, antibody CM1.1 which was also used as an antigenic benchmark mAb itself, was the most effective MR191 competitor of all the antibodies tested, knocking out more than 99% of MR191 binding when pre-incubated with RAVV GPΔMuc, better than MR191’s competition against itself (Fig. 7A and B). Remarkably, out of the 10 variants that effectively competed more than 65% of MR191’s binding to GP, only four effectively competed with benchmark antibody CM1.1, namely, CM2.2, CM3, CM4, and CM2.1 (Fig. 7A and B). The remaining five MR191 competitors, CM5, CM6, CM7, CM8, and CM9, competed to a lesser degree or not at all with CM1.1, suggesting that CM1.1 binding to GP was more difficult to block than MR191’s or that the MR191 epitope coincided more directly with these five antibodies. While antibodies CM1.2 and CM2.3 were not effective at competing with either MR191 or CM1.1, these two variants, as noted above, exhibited signs of biochemical instability and were weak binders to GP (Fig. 7A, B, and 4B).

For antigenic benchmark antibody CM10, whose epitope mapped to the GP2 wing (Fig. 6), the assay revealed as expected that pre-incubation of GP with antibodies CM11.1 and CM12.1 reduced CM10 recognition of GP by 94% and 91%, respectively (Fig. 7A and B). Antibodies CM11.1 and CM12.1, like CM10, bound denatured GP by Western blot analysis and their epitopes mapped to the same overlapping residues within the GP2 N terminus as CM10’s (Fig. 6). Our binding competition assays thus confirmed that all three antibodies, CM10, CM11.1, and CM12.1 fell within the same binding competition group and recognized a common overlapping GP2 wing epitope in the context of the GPΔMuc ectodomain (Fig. 7A, B, and E). Two lineage mates of CM11.1 and CM12.1, CM11.2 and CM12.2, respectively, were not evaluated in these assays but were confirmed in a parallel study to target the same GP2 epitope (B. Janus, G. Ofek, unpublished results). None of the other antibodies in the panel successfully competed with CM10 for GP recognition (Fig. 7A and B).

For antigenic benchmark antibody CM13, whose epitope mapped to a contiguous region on GP1 in the vicinity of the RBR, the binding competition assays revealed that three other antibodies fell within its antigenic competition group: CM14, CM15, and CM16. Pre-incubation of RAVV GPΔMuc with any one of these three antibodies blocked CM13 recognition of GP by 40%–52% (Fig. 7A and B). None of these mAb competitors recognized denatured GP by Western-blot analysis, suggesting their epitopes were conformational in contrast to that of CM13. We note that antibody CM21, which we could not map by pepscan analysis but appears to recognize denatured GP1 by Western-blot analysis, blocked CM13 binding to GP by ~25%, indicating possible overlap in their epitopes (Fig. 6, 7A, B, and E).

Our binding competition assays also revealed that pre-incubation of RAVV GPΔMuc with several antibodies in the panel could enhance benchmark antibody binding to GP. In particular, mAbs CM1.1 and CM2.2, within the RBR-directed antigenic competition group, enhanced the binding of benchmark mAb CM13 to RAVV GPΔMuc by ~20% (Fig. 7A and B). Since antibody cooperativity in virus neutralization has been reported for orthoebolaviruses, further studies will be necessary to assess whether cooperativity in binding observed here also translates into cooperativity in virus neutralization (56, 57).

The seven remaining GP-reactive antibodies in the panel, CM17 through CM23 did not robustly fall into any of the four antigenic competition groups tested (Fig. 7A and B). These antibodies may target epitopes on RAVV GPΔMuc distinct from those of the benchmark antibodies, although we cannot exclude the possibility that the absence of effective competition is a result of insufficient binding affinity to RAVV GPΔMuc as opposed to complementary recognition.

### Analysis of CM1.1 and CM2.1 recognition of RAVV GPΔMuc by negative stain electron microscopy

To confirm the GP binding targets of the two antibody lineages that exhibited the highest potency of virus neutralization, CM1 and CM2, we analyzed their recognition of RAVV GPΔMuc by negative stain electron microscopy (NSEM). Toward this end, fragments of antigen binding (Fabs) of CM1.1 and CM2.1 were expressed and individually complexed with recombinant RAVV GPΔMuc protein. Each complex was applied to EM grids and stained with uranyl formate prior to imaging on a Talos Arctica (200 kV) system. Data processing and 2D classification were performed using RELION (49). As shown in Fig. 7C and D, 2D classes generated for the complexes of CM1.1 Fab and CM2.1 Fab with RAVV GPΔMuc yielded particles with either one or two Fabs bound at the apex of GP. Observed structures were consistent with those observed for antibodies that target the predicted orthomarburgvirus GP RBR, specifically antibodies MR191 and MR78 (Fig. 7E; Fig. S6) (11, 58).

### MARV-VSV pseudovirus release

Previous reports indicate that some antibodies that target the mucin-like domain on MARV GP can inhibit virus release from host cells by leading to aggregation of viral particles on the host cell surface (15). Although none of the antibodies in our panel mapped to the mucin-like domain on GP, we nonetheless sought to assess whether selected antibodies could inhibit viral particle release. Toward this end, we implemented an assay that measured the effect of antibodies on MARV-VSV pseudovirus titers released into cell culture supernatants when produced in the presence of individual antibodies (Fig. S7). We selected non-RBR, non-neutralizing antibodies that bound tightly to GP for testing in this assay to avoid conflation with inhibition of MARV-VSV entry. Antibodies tested included representatives of all three GP2-wing directed mAb lineages (CM10, CM11.1, and CM12.1), mAb CM13, and mAbs CM20 and CM21 that did not fall into any binding competition group. Orthoebolavirus GP-specific mAb CA45 was assessed in parallel as a negative control. As shown in Fig. S7, the effects of the antibodies tested in this assay varied. When analyzed using an unpaired two-tailed *t*-test, mAb CM20 exhibited a statistically significant inhibition of MARV-VSV release relative to the no-antibody controls, with a *P*-value of 0.0072 (Fig. S7). The effects of the other mAbs on MARV-VSV release were not statistically significant. Further studies will be necessary to assess the mechanisms underlying these results and to confirm whether similar results hold when tested against more native filoviral particles.

### Cross-filovirus GP recognition

**Fig 8:**
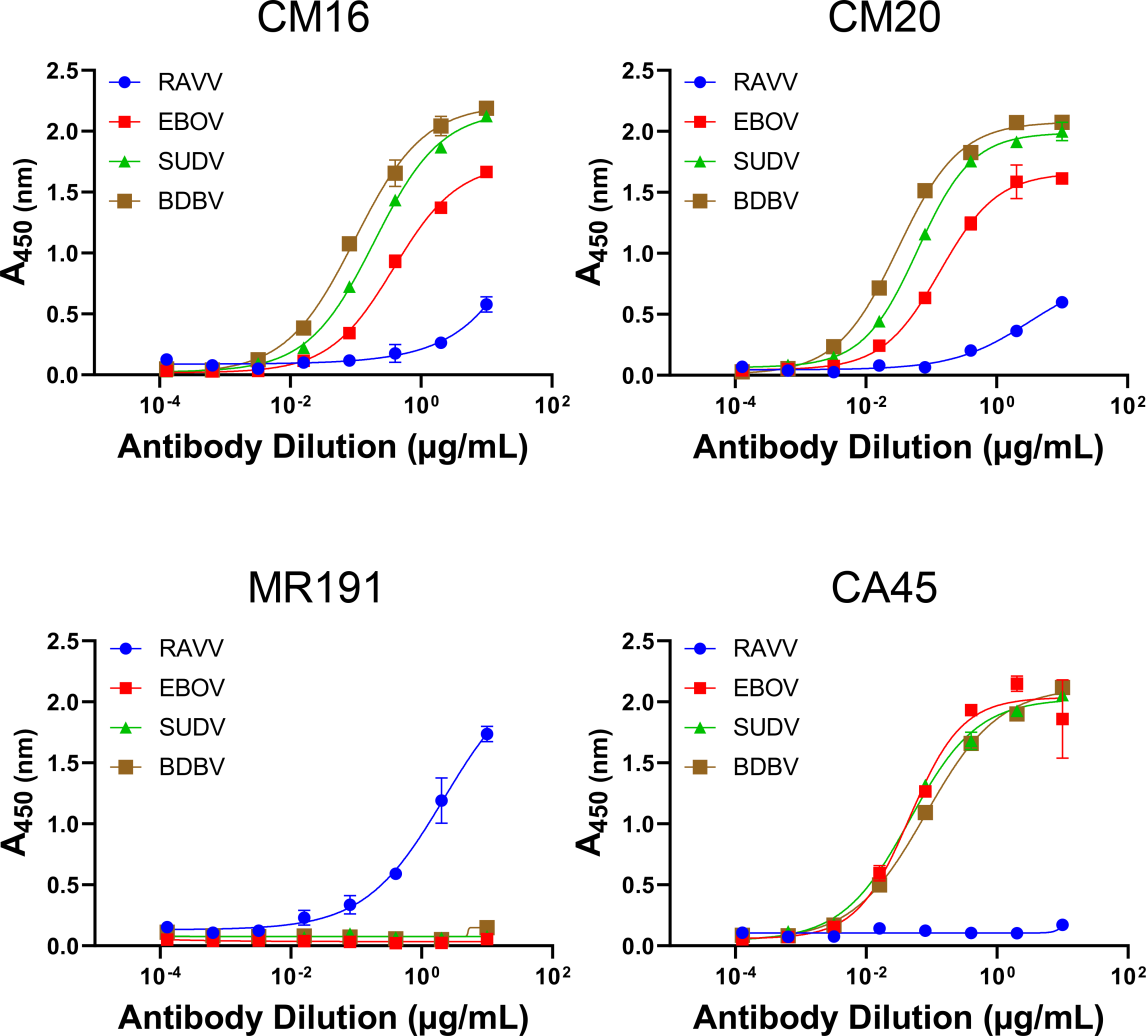
Cross-filovirus GP recognition. ELISA binding profiles of antibodies CM16 and CM20 to RAVV, EBOV, SUDV, and BDBV GPΔMuc proteins. Orthomarburgvirus-specific mAb MR191 and orthoebolavirus-specific cross-reactive mAb CA45 were analyzed as controls. Shown are means of technical duplicates with error bars indicating standard deviation. Results are of representative experiments repeated at least three times.

Since animal NHP1 received multivalent immunizations that included SUDV- and EBOV-based antigens, we next assessed whether any of the 28 antibody lineages could recognize orthoebolavirus GPs as well. Toward that end, all mAbs in the panel were tested for recognition of recombinant EBOV GPΔMuc by ELISA. While a majority of the mAbs had weak to undetectable binding (not shown), two of them—CM16 and CM20—did exhibit measurable binding (Fig. 8). Subsequent assessment of CM16 and CM20 for recognition of other orthoebolavirus GPs, namely SUDV and BDBV GPΔMuc, revealed that both antibodies recognized SUDV and BDBV GPΔMuc equally well if not better than their recognition of EBOV GPΔMuc (Fig. 8). CM16 and CM20 binding to orthoebolavirus GPs was similar to that observed for the control antibody CA45, although their recognition of RAVV GPΔMuc trailed that of the MR191 control (Fig. 8).

Despite only weak or undetectable neutralization of MARV GP pseudoviruses by mAbs CM16 and CM20, in view of their cross-filovirus GP recognition, we assessed their neutralization of orthoebolavirus psuedoviruses. Neither CM16 nor CM20 exhibited detectable neutralization of EBOV, SUDV, or BDBV pseudoviruses, suggesting that they either targeted a conserved epitope that does not confer inhibition of entry or that other features rendered them ineffective in preventing viral entry at the concentrations used in these assays (not shown). Taken together, our results confirmed that the multivalent prime-boost immunization regimen given to NHP1 led to the successful induction of monoclonal antibodies with cross-filovirus reactive breadth.

## DISCUSSION

In the present study, we explored a multivalent filovirus prime-boost immunization approach in nonhuman primates to induce immunological breadth against filovirus glycoproteins for downstream mAb isolation. All animals were primed exclusively with MARV GP-based antigens to ensure responses against orthomarburgviruses would effectively take hold in the absence of exposure to GP antigens of other filoviruses. Subsequent repetitive boosting with MARV immunogens alongside SUDV and then EBOV immunogens was not only aimed to induce autologous responses against all three species but also to induce cross-reactive heterologous antibody responses against conserved regions on GP both within and across the *Orthoebolavirus* and *Orthomarburgvirus* genera. Indeed, all animals in the study successfully developed both autologous and heterologous antibody titers against multiple filovirus species. Using PBMCs from the animal that exhibited the highest titers of serum antibody responses against Marburg virus GP, we isolated and characterized a novel panel of cross-reactive GP-specific mAbs.

Our analysis revealed that roughly a third of the antibodies in the panel mapped to the RBR on GP1, including two lineages—CM1 and CM2—that exhibited potent MARV pseudovirus neutralization. To our knowledge, other than the panel of antibodies isolated from a human survivor of MARV infection and bioinformatically identified homologs thereof, these antibodies are the only other cases of RBR-directed orthomarburgvirus neutralizing antibodies that have been reported to date, and represent the first such nAbs induced and isolated from animal immunizations (11, 16). Further studies will be necessary to elucidate whether the induction and isolation of the CM1 and CM2 lineages was a result of the multivalent prime-boost immunization regimen administered to NHP1, or alternatively, to features of the downstream antigen-specific single B-cell sorting pipeline that was used to isolate the panel as a whole, including possibly the heterologous RAVV GPΔMuc probe itself.

In addition to the RBR binding competition group, three antibody lineages in the panel, CM10, CM11, and CM12, mapped to the GP2 wing protective region (17, 18). CM10, CM11, and CM12 all recognized the same continuous epitope within this region, spanning residues 450–464, that partially or fully overlapped with epitopes of protective mAbs 30G4 and MR228 (17, 18). GP2 residues 450–464 are fully conserved across all MARV isolates but differ within RAVV GP at 5 of 15 residue positions. Since CM10, CM11, and CM12 were all solely induced by MARV-based GP antigens, their cross-reactive recognition of RAVV GP likely relies on conserved residue positions within this region or on accommodation of sequence variation.

The third antigenic competition group that we identified mapped to an epitope on GP1 that spanned residues 61–75, a partially conserved region across filoviruses. This region was previously identified as the target of a pan-filovirus reactive murine antibody m21D10 (29). Four antibodies in the panel fell within this antigenic group, CM13, CM14, CM16, and CM16. While mAb CM13 bound a peptide spanning this region and to denatured GP, mAbs CM14, CM15, and CM16 did not bind this peptide nor did they recognize denatured GP, suggesting they target conformational or complex epitopes that overlap but are nonetheless distinct from that of CM13.

An additional goal of the present study was to use multivalent prime-boost immunization to induce mAbs with pan-filovirus reactivity. Two mAbs in the panel, CM16 and CM20, were found to possess pan-filovirus reactivity and recognized orthomarburgvirus as well as multiple orthoebolavirus GPs, including of SUDV, EBOV, and BDBV. Remarkably, mAb CM16 fell within the CM13 binding competition group, whose epitope on GP1 overlapped that of pan-reactive murine antibody m21D10 (29), consistent with definition this region on GP1 as a pan-reactive target.

Lastly, we note that seven antibodies in the panel, including pan-filovirus reactive mAb CM20, could not be unambiguously mapped to any known site on GP, posing the possibility of additional antigenic targets on GP that have yet to be fully defined. Taken together, our study expands the available repertoire of mAbs directed against orthomarburgvirus GP, including novel neutralizing lineages targeting the RBR. It also advances the understanding of orthomarburgvirus GP antigenicity and determinants of antibody cross-reactivity. Further studies will be necessary to assess antibody protective breadth and efficacy in animal challenge models of filovirus infection.

## Data Availability

Data associated with this study are included in the article itself or are freely available upon request to Gilad Ofek (gofek@umd.edu). Requests for reagents can be directed to Gilad Ofek and will be made available upon an executed Materials Transfer Agreement.

## SUPPLEMENTAL MATERIAL

Supplemental figures jvi.00155-24-s0001.pdf Figures S1 to S7.

ASM does not own the copyrights to Supplemental Material that may be linked to, or accessed through, an article. The authors have granted ASM a non-exclusive, world-wide license to publish the Supplemental Material files. Please contact the corresponding author directly for reuse.
