# Supplementary material for: Macaque antibodies targeting Marburg virus glycoprotein induced by multivalent immunization
Source: J Virol. 2024 Jun 4;98(7):e00155-24. doi: 10.1128/jvi.00155-24 (PMC11329191; doi:10.1128/jvi.00155-24)
Supplement: Supplemental figures — Figures S1 to S7. [file jvi.00155-24-s0001.pdf]

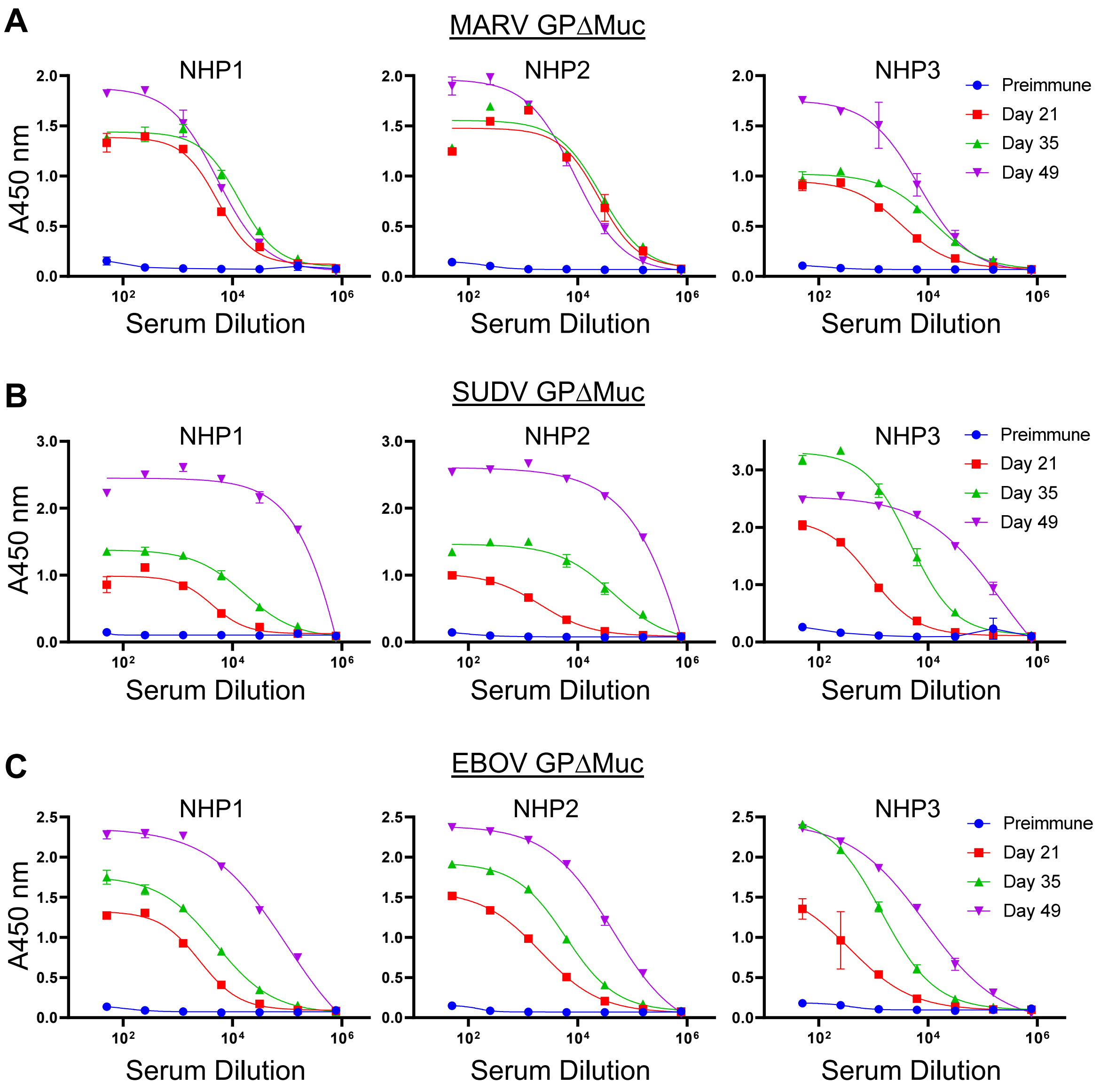

Figure S1. Serum antibody recognition of autologous GPs. ELISA binding profiles of serum bleeds from each animal taken on study days 0 (preimmune), 21, 35, and 49 to recombinant MARV GPΔMuc (**A**), SUDV GPΔMuc (**B**), and EBOV GPΔMuc (**C**). Shown are means of technical duplicates with error bars indicating standard deviation. Results are of representative experiments repeated two or more times.

A

MARV GPΔMuc

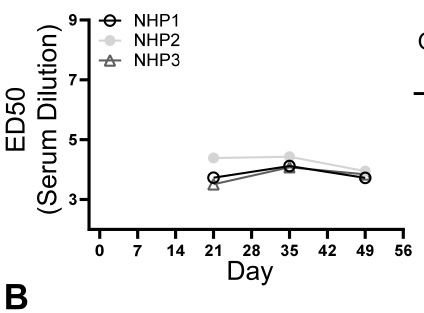

MARV GPΔMuc	ED50 (Serum Dilution) Day 21			Day 35			Day 49		
	ED50	LogED50	LogSE	ED50	LogED50	LogSE	ED50	LogED50	LogSE
NHP1	5333	3.727	0.049	13173	4.120	0.058	5276	3.722	0.041
NHP2	24529	4.390	0.135	26906	4.430	0.158	8898	3.949	0.046
NHP3	3224	3.508	0.057	12039	4.081	0.068	6881	3.838	0.078

B

SUDV GPΔMuc

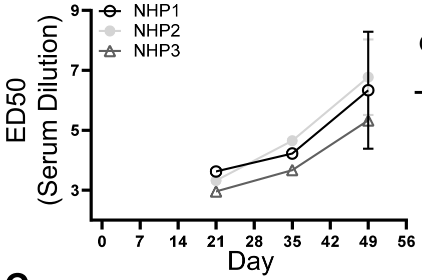

SUDV GPΔMuc	ED50 (Serum Dilution) Day 21			Day 35			Day 49		
	ED50	LogED50	LogSE	ED50	LogED50	LogSE	ED50	LogED50	LogSE
NHP1	4239	3.627	0.120	16730	4.224	0.045	2177408	6.338	1.956
NHP2	2084	3.319	0.039	44589	4.649	0.133	5990506	6.777	1.261
NHP3	914.2	2.961	0.025	3.673	0.047	1.261	213765	5.330	0.175

C

EBOV GPΔMuc

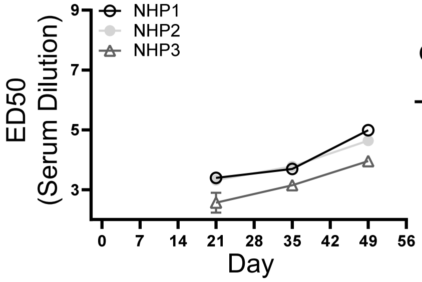

EBOV GPΔMuc	ED50 (Serum Dilution) Day 21			Day 35			Day 49		
	ED50	LogED50	LogSE	ED50	LogED50	LogSE	ED50	LogED50	LogSE
NHP1	2493	3.397	0.035	4969	3.696	0.043	98166	4.992	0.148
NHP2	2101	3.322	0.024	6047	3.782	0.017	43753	4.641	0.040
NHP3	371.6	2.570	0.332	1416	3.151	0.023	9032	3.956	0.046

Figure S2. Serum antibody binding ED50s to autologous GPs across study days 21, 35, and 49. Progression of serum ELISA 50% binding effective dilutions (ED50s) across study days 21, 35, and 49 to recombinant MARV GPΔMuc (**A**), SUDV GPΔMuc (**B**), and EBOV GPΔMuc (**C**). Binding ED50, logED50, and logSE were calculated in Graphpad Prism using the plots shown in Fig. S1. Error bars represent logSE (log Standard Error).

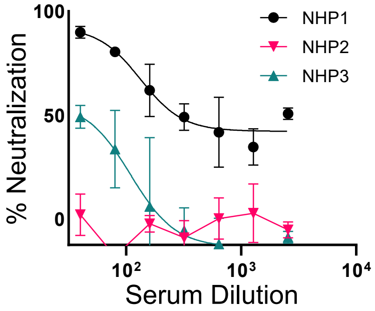

Figure S3. NHP1, NHP2, and NHP3 day 49 serum neutralization of heterologous marburgvirus RAVV GP MLV psuedoviruses. Shown are means of technical duplicates with error bars indicating standard deviation. Results are of a representative experiment repeated three times.

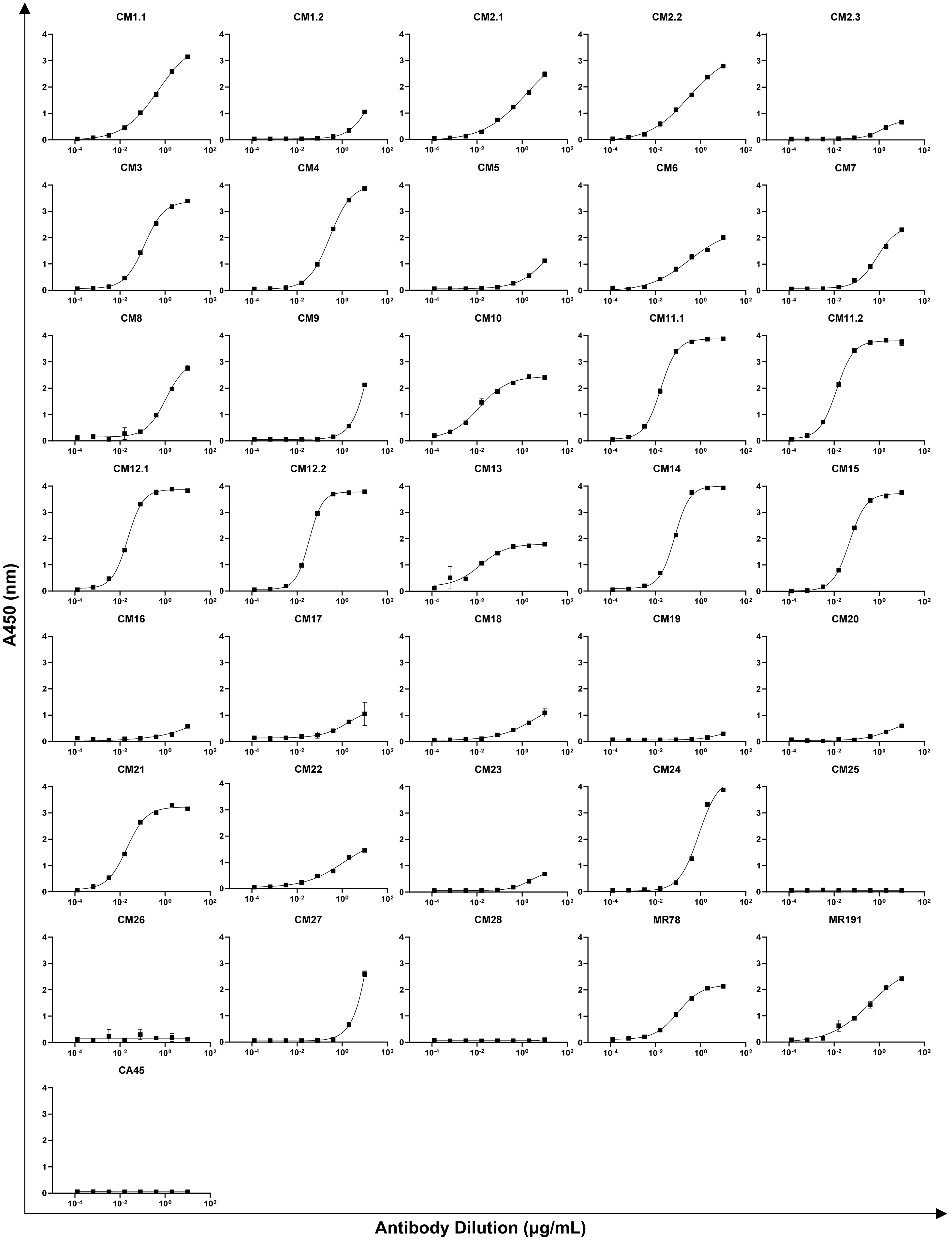

Figure S4. Antibody ELISA binding profiles to RAVV GPΔMuc. ELISA binding profiles of each individual antibody to RAVV GPΔMuc. Shown are means of technical duplicates with error bars indicating standard deviation. Results are of representative experiments performed at least two times for antibodies that exhibited detectable binding.

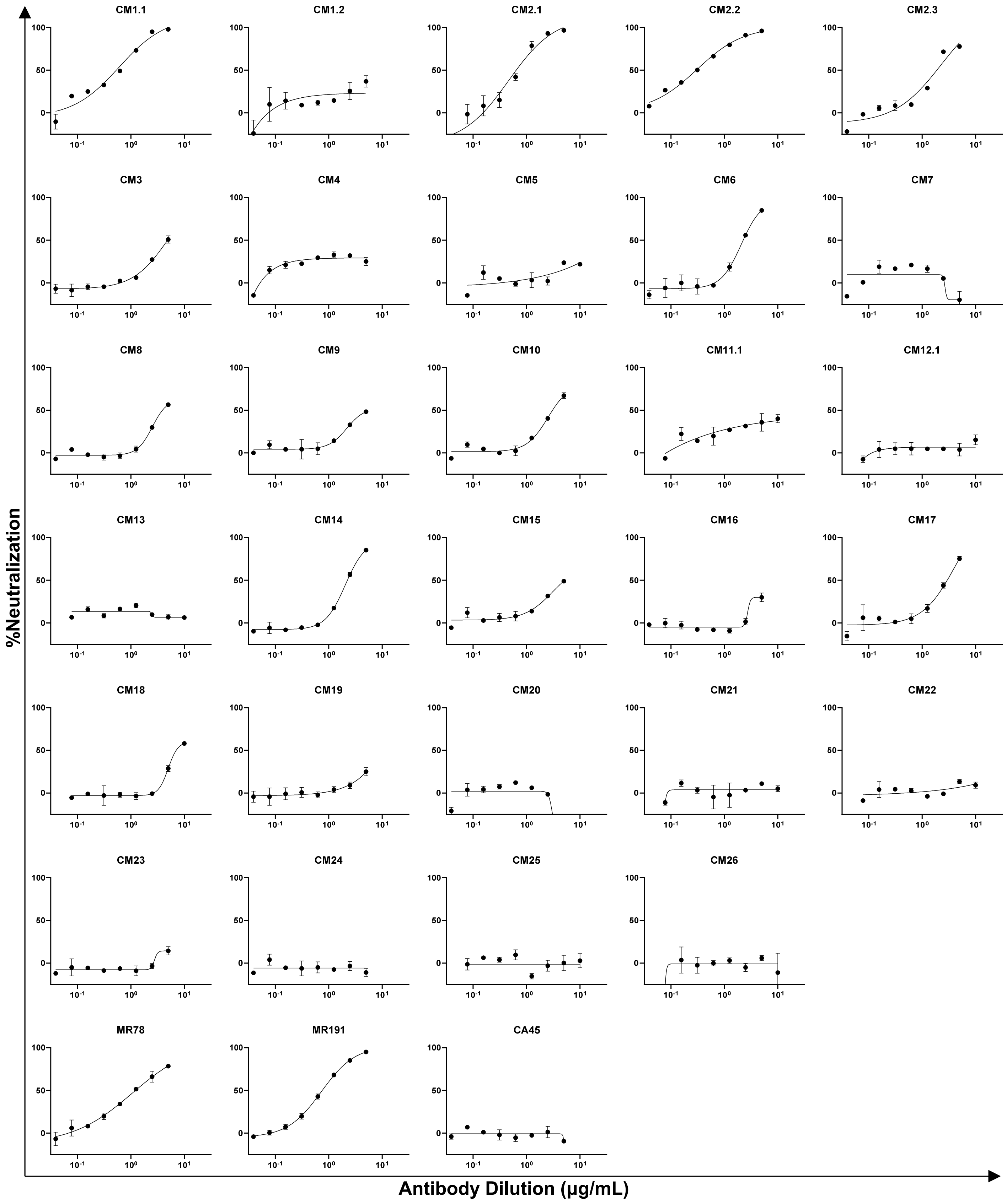

Figure S5. Antibody neutralization of Musoke MARV-MLV pseudoviruses. Neutralization profiles of each individual antibody against MARV-GP MLV pseudoviruses. Shown are means of technical duplicates with error bars indicating standard deviation. Results are of representative experiments performed at least two times for antibodies that exhibited detectable neutralization.

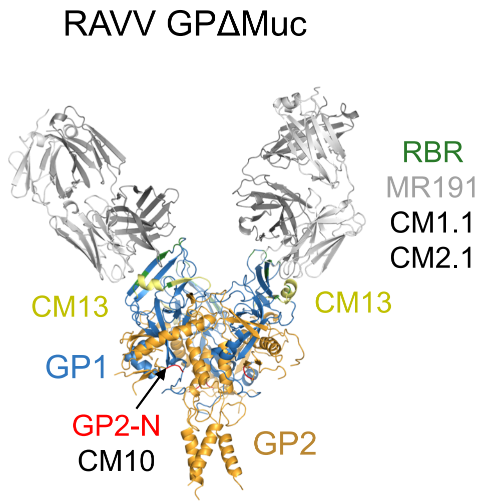

Figure S6. Cartoon representation of the crystal structure of RAVV GPΔMuc in complex with RBR-directed neutralizing antibody MR191 (PDB ID 6BP2). GP1 and GP2 are colored blue and orange, respectively, and MR191 is colored gray.

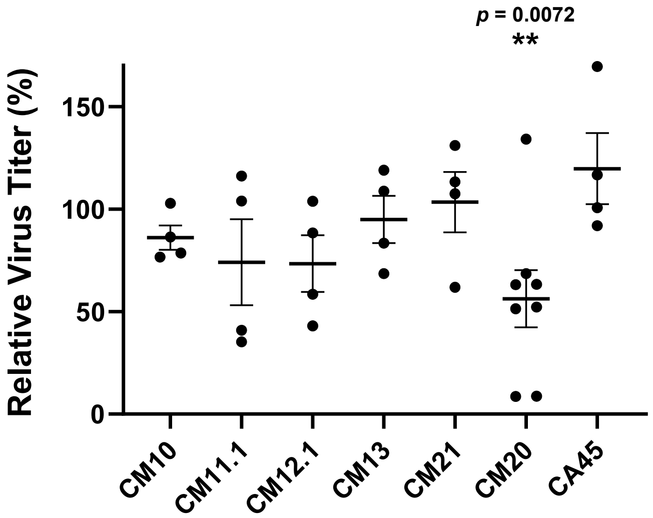

Figure S7. MARV pseudovirus release in presence of antibodies. MARV-VSV psuedovirus titers released into cell culture supernatants when produced in the presence of listed antibodies, shown as a percentage relative to titers released in the absence of antibodies. Values are means across two independent experiments, with 4-8 technical replicates per antibody. Error bars indicate standard error of the mean. Ebolavirus specific antibody CA45 was used as a negative control.
